# To the Physicians of Vermont

**Published:** 1915-02

**Authors:** 


**TO THE PHYSICIANS OF VERMONT:**

Owing to the discontinuance of the Vermont Medical Monthly, we feel that an opportunity presents itself for extending the field of local representation of the medical profession in regard to news items, discussion of local problems and adequate facilities for the publication of original articles. It is economically impossible to conduct a journal for a locality of such population as the State of Vermont or the City of Buffalo although a medium thus strictly local, may be conducted as a matter of public spirit, expressed co-operatively or privately. Conversely, our policy has been against active competition with any existing local medium.

You will note from the Associate Staff on the front cover, that our field now includes western and central New York, equivalent to about one-twentieth of the entire country or to about two and a half average states. Without, as yet having formally arranged for editorial representation, we also serve adjoining territory, as a local medium. Samples of the February and March issues will be distributed so as to cover the medical profession of Vermont. Please note carefully the general contents, standing headings, etc. If satisfied with our good faith in offering to serve as a local medium and convinced of the necessity of such a medium, please send your subscription to cover Vol. 71, August, 1915, to July, 1916, and the remaining numbers of the current volume will be sent free. Incidentally, please note that this is the third medical journal of the western continent in age. Editorial representation, proper designation of the enlarged field of the Journal and subdivision of departments will be arranged as soon as possible. Suggestions and news items, original articles—not including ''addresses'' and meditations—are requested, irrespective of subscription. The attention of officers of medical societies is especially called to the general heading on the editorial page and to that of the department of Society Meetings.

A prompt reply is requested, that we may determine whether or not the profession of Vermont desires a local organ. If the response is not sufficient to warrant editorial representation and adequate local service, subscriptions may be canceled and there will be ample time to do so before the subscription remittance is due.

